# Direct-to-Consumer Nutrigenetics Testing: An Overview

**DOI:** 10.3390/nu12020566

**Published:** 2020-02-21

**Authors:** Matteo Floris, Antonella Cano, Laura Porru, Roberta Addis, Antonio Cambedda, Maria Laura Idda, Maristella Steri, Carlo Ventura, Margherita Maioli

**Affiliations:** 1 Department of Biomedical Sciences, University of Sassari, Viale San Pietro 43/b, 07100 Sassari, Italy; antonella.cano@gmail.com (A.C.); l.porru@studenti.uniss.it (L.P.); antonio.cambedda85@gmail.com (A.C.); 2 Institute for Genetic and Biomedical Research, National Research Council, traversa La Crucca 3, 07100 Sassari, Italy; m.laurai@yahoo.it; 3 Department of Chemistry and Pharmacy, University of Sassari, Via Muroni 23/A, 07100 Sassari, Italy; raddis@uniss.it; 4 Institute for Genetic and Biomedical Research, National Research Council, SS 554 Km 4,500, Monserrato, 09042 Cagliari, Italy; maristella.steri@irgb.cnr.it; 5 Laboratory of Molecular Biology and Stem Cell Engineering, National Institute of Biostructures and Biosystems–Eldor Lab, Innovation Accelerator, CNR, Via Piero Gobetti 101, 40129 Bologna, Italy; ventura.vid@gmail.com; 6 Center for Developmental Biology and Reprogramming-CEDEBIOR, Department of Biomedical Sciences, University of Sassari, Viale San Pietro 43/b, 07100 Sassari, Italy

**Keywords:** genetic, nutrigenetics, **direct-to-consumer genetic testing** (DTC-GT), nutrigenomics, diet, nutrition

## Abstract

At present, specialized companies offering genetic testing services without the involvement of clinicians are growing; this development is a direct consequence of the significant decrease in genotyping and sequencing costs. Online companies offer predictions about the risk of developing complex diseases during one’s life course, and they offer suggestions for personal lifestyle. Several companies have been created that provide nutrigenetics services; these companies suggest dietary indications—a central issue in the prevention and etiopathogenesis of specific diseases—based on one’s personal genetic background. Dietary patterns are defined on the basis of a limited set of genetic markers. In this article, we analyze the online nutrigenetics services offered by 45 companies worldwide, to obtain an overall picture of the costs, the types of nutritional traits considered and the level of scientific precision of the services proposed. Our analysis clearly highlights the need for specific guidelines, in order to ensure a set of minimum quality standards for the nutrigenetics services offered to the customer.

## 1. Introduction

In recent years, several companies have started selling DNA testing kits via the internet directly to consumers (DTC) [1]. Such “consumer genetics” companies communicate genetic test results to the customer without medical supervision and offer a wide range of predictions about the personal risk for developing common diseases including cancer, autoimmune or cardiovascular diseases. Overall, “direct-to-consumer genetic testing” services, or DTC-GT, are supposed to guide the user toward more informed decisions about lifestyle choices. Usually, the genetic test starts with an online order of a kit by a consumer, necessary for the collection and delivery of a saliva sample; the companies then extract the consumer’s DNA and assess the presence or absence of specific genetic variants known to be associated with (for example) an increased disease risk, or with the regulation of a trait of interest and various health conditions. Interestingly, several companies have focused their service toward health-related outcomes such as fitness (e.g., performance and injury tests) [2], pharmacogenetics (e.g., personalized treatment) [3] and nutrigenetics (e.g., weight control, and food intolerance and sensitivity) [4].

The continuous growth of DTC-GT companies is fueled by both the ongoing dramatic drops in DNA sequencing and genotyping costs, and by the availability of a wealth of human genetic variation data. Furthermore, the Human Genome Project (HGP, a 13-year project begun formally in 1990 and coordinated by the National Institutes of Health and the U.S. Department of Energy) [5] in 2003 provided researchers with the full sequence of the human genome (“reference genome sequence”), which allows researchers to define genetic variants (differences in the sequence of DNA among individuals) and to study their functional consequences.

The most common class of genetic variants, termed single nucleotide polymorphisms (SNPs), is represented by a single nucleotide change with respect to the reference sequence (for example, a cytosine C is replaced by a thymine T) at a given position of the genome. So far, scientists have found more than 600 million SNPs in human populations around the world [6]. The second most abundant class of genetic variants is insertions and deletions (INDELs), where a person’s DNA sequence at a given position of the genome has more (insertions) or less (deletions) nucleotides with respect to the reference sequence.

SNPs and INDELs, both clinically relevant as well as neutral genetic variants, are registered in a free public archive, the Single Nucleotide Polymorphism Database (dbSNP) [7]. A specialized subset of dbSNP entries is collected in ClinVar [8], a freely available, public archive of human genetic variants with proven or suspected clinical relevance. A unique identification tag—the so-called “rs identifier” or “rs ID”—is assigned to each genetic variant stored in dbSNP. For example, the ID rs1801280 corresponds to a genetic variant that changes the aminoacidic sequence of the protein encoded by the gene NAT2 (N-Acetyltransferase 2, see [9]). Rs identifiers are particularly useful when searching for information about a variant, because they are unambiguous, unique and stable; in contrast, descriptive names of genetic variants (based on their genetic position or on amino acid changes) can be more ambiguous, depending for example on the version of the human genome used as a reference.

The majority of common genetic variants have little or no effect on health or development. However, in certain special cases, they can influence an individual’s response to certain drugs [10], or increase the risk of developing a complex disease such as type 1 diabetes [11] or Alzheimer’s disease [12].

Genome-wide association studies (GWAS) are a relatively recent and effective way to identify genetic variants and, consequently, genes associated with disease risk and human quantitative traits (such as body mass index or blood levels of a metabolite). GWAS can be based on SNP array data, or on the full genomic sequence; in the second case, variants can be obtained either by directly sequencing individuals or statistically inferring (by “imputation”) the unobserved genotypes in array data from the reference genome [13]. Large sample sizes are required to accurately estimate the allelic frequencies and, consequently, to obtain a statistically robust finding. When a large number of genetic markers are tested in the same experiment, the results need to be corrected for multiple testing; for GWAS in particular, results are considered reliable when associations achieve an accepted genome-wide statistical significance threshold, conventionally represented by a *p*-value < 5 × 10^−8^. Despite the success of GWAS in identifying variant–trait associations and their application to clinical analysis, several limitations need to be taken into account when interpreting the results. One of the most relevant limitations is that, in each genetic region, multiple variants can be correlated (they are in “linkage disequilibrium”): on one hand, this facilitates the identification of the association but, on the other hand, makes it difficult to identify the “causal” variant(s), i.e., the variant effectively responsible for the signal. This means that the effective biological effect is unknown. Also, several studies have demonstrated that not only allele frequencies but also biological effects can be different in populations of distinct ancestries: this implies a further level of complexity in identifying the underlying biological mechanisms, which can be different for example in Europeans with respect to Asians or Africans [14].

At present, the large success of GWAS is represented by a large number of common variants associated with complex diseases that are freely available in specific databases, such as the GWAS Catalog (https://www.ebi.ac.uk/gwas/), in particular for neurological, immunological and cardiovascular diseases [15,16]. In addition, a wide number of genetic variants associated with clinical, biochemical and anthropometric traits are available. For example, as of 16 December, 2019, the GWAS Catalog contained 4346 publications and 166,103 genetic associations.

In a recent paper, Zenin et al. [17] identified 35 traits with significant and large genetic correlation to health-span, which can be classified into four clusters: (1) sociodemographic factors, lifespan, smoking and coronary artery disease; (2) high-density lipoprotein (HDL)-related traits; (3) obesity-related disease and body mass index (BMI); and 4) type 2 diabetes-related traits. Therefore, there is a strong genetical correlation between health-span, as a morbidity-free life period, personal history and life-traits, which might be controlled by physiological processes such as nutrition.

Nutritional genomics can be considered a branch of personalized medicine [4]. It is widely known that many genetic variants can influence the body’s metabolism and responses to nutrients (nutrigenetics) but, on the other hand, nutrients themselves can also modulate gene expression (nutrigenomics) [18]. Nutrigenetics and nutrigenomics might therefore be defined as two alternative approaches to nutritional genomics.

The identification of genetic variants associated to a specific diet-related disease or to a particular response to food will allow the elaboration of dietary strategies and population-wide dietary recommendations [4]. A nutritional genomic approach is reliable only for some conditions such as monogenic diseases—e.g., phenylketonuria and galactosemia (and many other similar diseases)—caused by alterations of a single gene product that can be specifically tested [19]. However, this is typically not the case for complex traits (i.e., obesity, vitamin levels) and diseases (i.e., type 2 diabetes, cardiovascular disease), which result from a combination of genetic and environmental factors. A key measure of the importance of genetic factors is the heritability (h2), the proportion of phenotypic variation of a trait that is due to genetic variation [20]. It should be noted that heritability does not represent the proportion of a trait determined by genes. Rather, a heritability of, for example, 0.7, means that 70% of the variability in the trait in a specific population is due to genetic differences among the individuals in that population. Each estimate of the trait heritability is specific to a population in a specific environment, and its value, depending on the phenotypic variability, can change as the environmental conditions change [21,22]. An interesting example is BMI, whose heritability in higher-risk obesogenic home environments has been estimated to be about 86%, more than double that for those living in lower-risk obesogenic home environments (39%) [23].

When traits and diseases are highly heritable (i.e., monogenic traits and disorders determined by one or a few variants), genetic testing will be accurate and very predictive. In contrast, when traits and diseases are only partially influenced by genetic factors and the heritability is low, the predictive ability of tests that consider only a single genetic variant will never be very accurate [24,25]. Simply put, in case of complex traits and diseases, having a genetic variant will not mean developing a certain phenotype.

The complexity of these concepts means that a generic user does not have the tools to understand the truthfulness and reliability of these tests, unless direct-to-consumer genetic-testing (DTC-GT) companies declare in a transparent way the origin of the predictors used and the reliability of their advice.

Despite the complexity of these arguments, according to a MIT Technology Review article published in February 2019, “more than 26 million consumers added their DNA to four leading commercial ancestry and health databases” [26]. A recent analysis of DTC-GT companies performed by KPMG International assessed that this market is supposed to grow to over one billion USD by 2020 [27], probably being around six billion USD by 2028 [28].

A recent survey of a sample of the European population showed that 30% of individuals are strongly convinced that personalized genetic counseling would be effective in improving their eating behavior and felt that paying for such a service seems to be incorrect [29].

Within this context, the aim of the present work is to provide an overview of nutrigenetics online services, in order to understand what types of nutritional traits are analyzed by the various companies, and what kinds of information are applied to support decisions and their degree of clearness. In particular, the availability of unambiguous indications about the genes and genetic variants used for nutrigenetics predictions is investigated.

## 2. Methods

### 2.1. Identification of Companies Offering Nutrigenetics DTC-GT

In the first phase of our study, through a web engine search we identified companies that offer DTC-GT through a website. The following keywords were used for searching: ‘nutrigenetics test’, ‘DNA and health’, ‘diet nutrigenetics’ and ‘nutrigenetics DNA kit’. For the purposes of the study, we considered only companies that appeared on the first ten pages of the online search results. The investigation took place between 15 January and 30 March 2019.

Of the companies here identified, we focused only on those explicitly offering DNA tests based on genes and variants involved in the genetic regulation of nutritional traits, which can be useful for the prevention of metabolic diseases and adjustable with specific dietary strategies. Therefore, we excluded companies offering DNA testing for diseases and/or other services beyond our target, such as ancestry or fitness. In the second phase of our study, we excluded companies providing DNA test services for hospital or specialized personnel, multinational selling kits to specialists or specialized centers, and nutritionist or biochemical analysis center web pages. Companies’ names were then anonymized by removing commercial names and using a random numerical identifier. When companies used different names for the same trait, we adopted an arbitrary unique trait name.

### 2.2. Analysis of Genes and Variants

For each of the 45 companies, we collected the information about genes and variants considered for the genetic profiling of the consumers. When available, the dbSNP identifiers [7] were used to query the GWAS Catalog [15] and Clinvar database [8].

## 3. Results

### 3.1. Companies That Offer Direct-to-Consumer Genetic Testing in the Field of Nutrigenetics

Our broad online investigation led to the identification of 63 companies that offered health, lifestyle and wellness services. Eighteen of these did not provide DTC-GT services and were excluded from our analysis. Forty-five companies, specifically providing nutrigenetics DTC-GT, were included in the study (Table 1).

**Figure 1:**
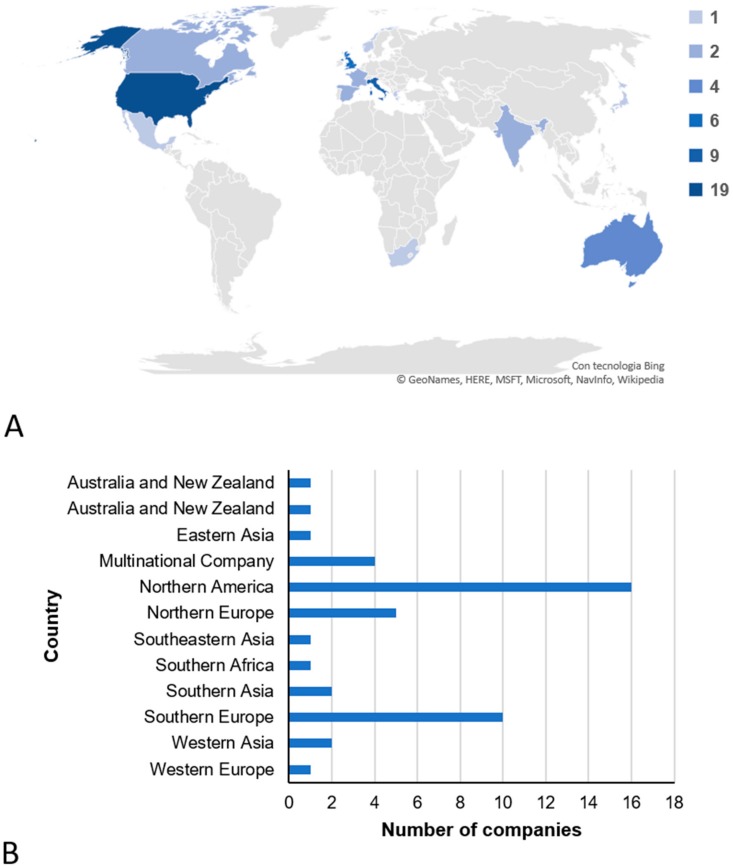
Geographical distribution of the selected nutrigenetics direct-to-consumer genetic testing (DTC-GT) companies. The legend reports the number of DTC-GT companies located in the represented countries (**A**). In graph (**B**), sub-continental classification of the 45 selected DTC-GT companies is shown. Multinational companies’ headquarters are located in several countries. N.A. = information not available.

Geographical analysis showed a world-wide distribution of the selected companies, although the highest numbers were observed in North America and in Europe, with 19 and 21 companies, respectively. Six companies were found in Asia and only two in Australia and New Zealand, whereas only one company was found in Central America, Southern Africa and Western Europe, respectively (Figure 1A). Four of the 45 are multinational companies, therefore they presented multiple localizations (Figure 1B).

Among the 45 selected companies, 41 provided a DNA kit either by online purchase or by an email or phone order. One company provided a DNA sampling kit, but the purchase/order modalities were unclear. Four companies did not explicitly indicate DNA kit buying information.

The DNA kit costs were indicated online only by 36 companies, whereas nine companies did not show any visible costs on their website. Along with useful protocols for DNA sampling, only 22 companies provided a sample report to consumers (Table 2).

### 3.2. Traits and Genetic Information

Quantitative and qualitative analysis was performed on the services, type and number of traits investigated (Table 3), along with the trait-associated genetic variants.

The most investigated traits belong to the category “micronutrients”. Thirteen companies provide DNA testing for genetic variants associated to vitamin D metabolism (29%), 10 companies to vitamin C, and nine companies to vitamin B12, vitamin A and vitamin B6. Among nutritional minerals, iron metabolism is the most tested (four companies).

Macronutrient metabolism is also investigated through genetic testing; in particular, a lipid metabolism test is provided by 13 companies (29%); carbohydrate metabolism and protein metabolism are tested by six companies and one company, respectively.

Food intolerance and food sensitivity are widely tested: lactose intolerance and caffeine sensitivity are investigated by 18 (40%) and 15 (33.33%) companies, respectively.

Interestingly, traits associated to physiological parameters and eating behavior are also studied, in particular levels of lipid profile (five companies) and weight management (12 companies, about 27%). A more detailed view of the data is summarized in Table S1.

**Figure 2:**
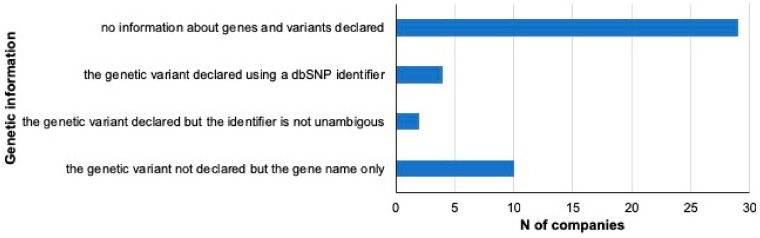
Characterization of the selected DTC-GT companies based on the genetic information provided. In the graph, the selected DTC-GT companies are classified based on the type of information on genes and variants analyzed that is provided to the consumer. N = number.

For a given gene, only four of the 45 selected companies provide information concerning the specific genetic variant investigated using an unambiguous *dbSNP* identifier. Ten companies do not indicate the genetic variant, but only the name of the gene associated, whereas four companies provide an ambiguous indication. On the other hand, 29 companies did not provide any information about the gene or the variant studied (Figure 2).

Overall, the resulting 16 companies consider 119 genes and 137 variants for their genetic profiling. Within the 137 variants, only 64 (47%) have a dbSNP identifier; for these dnSNP entries, we identified in the GWAS catalog a total of 480 genome-wide significant associations (*p* < 5 x 10^-8^) with 144 nutrition-related traits for only 36 variants. Notably, none of the companies clearly state the percentage of variance explained by the SNPs used for the risk assessment.

Furthermore, only 40 dbSNP variants out of 64 were found in the Clinvar database (of these, 19 were also found in the GWAS catalog and 28 are marked as entries of the “Online Mendelian Inheritance in Man” catalog - OMIM). All the details of genes and variants are available in Tables S2–S4.

**Figure 3:**
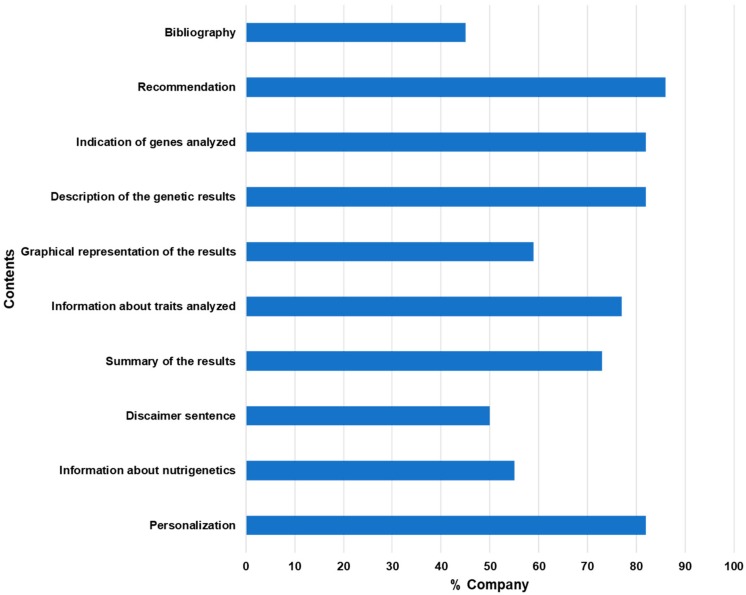
Analysis of the contents of the report examples provided by the selected DTC-GT companies. In the graph, the percentages of companies including specific contents in their report examples are represented in the graph.

Reports provided by the companies are heterogeneous about content and representation of results. Most of the example documents (82%) are presented as personalized reports with name, surname and sample ID. Twelve companies (55%) provide information about genes and SNPs. Only four companies cite GWAS and polygenic score risk. Overall, results are first represented in a summary of all traits studied and of the genetic results (73%) followed by trait-by-trait sections, where consumers are provided with information about traits analyzed (77%), gene names and variants (82%) and a graphical representation of the genetic results (59%) indicating whether the genotype is favorable or not, along with the increased, low, moderate or typical risk. Genetic results are clearly explained (82%) with one or more sentences that explain the significance, followed by recommendations (86%). Only 10 companies provide a bibliography to support the reported information. Eleven companies state a disclaimer sentence, indicating that the information provided is not for diagnostic or clinical use, and suggest consulting with a healthcare professional before making any major changes to diet (Figure 3).

## 4. Discussion

DTC-GT companies are specialized in providing nutritional advice based on an individual’s genetic background. Consumers, without a specific medical prescription, can obtain a set of information about their genetic predisposition to food-related disease or traits, by simply collecting saliva samples at home. Although the provided nutritional advice might lead the consumer to adhere to a better lifestyle, it should be properly explained and supported by scientific evidence. The present study focused on the analysis of the nutrigenetic services sold by 45 companies spread throughout the world. Interestingly, the most analyzed traits of nutritional interest were lactose intolerance and caffeine sensitivity. Since these intolerances are monogenic food-associated diseases, more reliable nutritional advice can be provided to consumers [4].

Predisposition to obesity, type 2 diabetes and cardiovascular disease is also widely analyzed by DTC-GT companies, although it is important to note that many factors that are not currently accounted for (such as multiple genetic variants with a general very small effect, environmental/lifestyle factors, and genetics–environment interaction) are involved in the potential development of these diet-related diseases.

We observed that the genetic analysis of lipid metabolism and weight management traits is commonly provided by about one third of the companies. Several genetic studies demonstrated an association between FTO (“fat mass and obesity-associated”) gene variants and obesity. For this reason, although little evidence supports the protective effect of specific nutritional protocols in individuals carrying FTO gene variants [4], FTO is the most investigated gene. Notably, the best-associated SNP in this gene (rs9939609) lies in intron 1 and explains <1% of the phenotypic variance of BMI and fat percentage in Europeans [30]; the minor allele increases BMI by 0.39 kg/m^2^ (or 1130 g in body weight) and is associated with a 1.20-fold increased risk of obesity [31]. This association has been confirmed across age groups and populations of diverse ancestry, although with different effects in different populations [31]. According to our analysis, three companies use only rs9939609, one company uses 5 SNPs and six companies do not declare the identifiers of the FTO genetic variants. It is also important to observe that, overall, 941 near-independent SNPs regulate BMI at a genome-wide significance level [32], explaining totally only 6.0% of its phenotypic variance. This means that, even though the FTO locus explains most of the interindividual variation in BMI, the ability to predict a person’s obesity risk based only on their FTO genotype is very limited.

Among the micronutrients, vitamins, such as vitamin D, are mostly investigated by the DTC-GT companies. Vitamin D is important in a wide range of physiological processes, and its deficit has been related to different chronic diseases and metabolic conditions, including obesity. Nonetheless, encompassing literature studies indicated only a faint association between gene variants acting in vitamin D metabolism and the obese phenotype [33], while the environment seems to play a major role [34,35,36]. The overall estimate of heritability of 25-hydroxyvitamin D serum concentrations attributable to the six susceptibility loci harboring genome-wide significant SNPs (recently identified in a large GWAS) is 7.5%, with statistically significant loci explaining 38% of this total [37]. These common variants tagged by GWAS chips therefore explain only a modest fraction of the overall variability in circulating 25-hydroxyvitamin D levels.

Considering scientific clearness, it should be noted that only 22 out of 45 companies provide a sample report on their website that clearly and comprehensively indicates the exact steps involved in the genetic analysis, with all the results provided to the consumer. Genetic risk level is frequently summed in a table, using an attractive and straight-forward color legend to indicate high, medium or low risk; however, allele variants are not always specified. Interestingly, the investigated traits are usually described and scientific references are linked, to give a general background to the consumer and to make the result interpretation easier.

However, the most outstanding fact is that only 16 companies out of 45 (about one third of the total) state which genes or which genetic variants are employed for nutrigenetic predictions. Moreover, only for 50% of the declared variants is an unambiguous code (in particular the dbSNP identifier) used. For this reason, for most companies it is difficult to understand exactly which genetic variants have been used to make predictions, and as a consequence it is very hard to interpret the reports and evaluate their scientific reliability.

In addition, of the 64 variants with the dbSNP identifier, only half of them are significantly associated at the genome-wide level with at least one trait of nutritional interest. This means that on average about 50% of genetic variants used for predictions show weak evidence of association with nutritional traits, and the chance that these variants might represent false signals of association is very high.

An interesting aspect is that predictions concerning traits for which dozens or hundreds of genetic associations (as in the case of body weight) are known, are made on the basis of few genetic variants.

Moreover, none of the companies exploit the use of powerful statistical tools such as polygenic risk scores (PRS, also known as risk profile scoring, genetic scoring, and genetic risk scoring). PRS combine multiple associated variants into a unique score by weighting their frequency in the population with their estimated impact on a trait [38]; they can be constructed for any complex genetic phenotype for which appropriate GWAS (or other robust association) results are available. PRS for susceptibility are promising tools to identify individuals at high risk who may be eligible for protective interventions, and their application could lead to more reliable information for consumers [24,25].

To the best of our knowledge, the present study provides new insight into DTC-GT companies’ characteristics and services in the nutrigenetic field. Considering the widespread proliferation of these services without medical advice, the evaluation of the scientific support and the understanding of the information provided could represent a great improvement.

## Figures and Tables

**Table 1:** Classification of the online company services. Direct-to-Consumer Genetic Testing (DTG-GT); Number (N°) of companies

Classification		N° Companies
**DTC-GT COMPANY**		**45**
**NOT DTC-GT COMPANY**		**18**
	DNA test services for hospital or specialized personnel	8
	Specialist web sites	3
	Multinational companies providing kit to specialists or specialized centers	2
	Biochemical analysis centres for patients	2
	Other	3

**Table 2:** Services and information provided by the selected companies.

Services and Information Provided		N° Companies
**DNA KIT**	**Provided**	**41**
	Purchase online	35
	Order by email or phone	5
	Not clear purchase modality	1
	**Not Provided**	**4**
**COST**	**Indicated**	**36**
	0-100 $	9
	100-200 $	13
	>200 $	14
	**Not Indicated**	**9**
**REPORT**	**Provided**	**22**
	**Not provided**	**23**

**Table 3:** Principal traits investigated by the selected DTC-GT companies. Number (N°) of companies

Category	Traits	N° of Companies
**Food intolerance**		
	Lactose intolerance	18
	Fructose intolerance	2
**Food Sensitivity**		
	Caffeine sensitivity	15
	Alcohol sensitivity	8
	Salt Sensitivity	5
	Sulphites sensitivity	1
	Nickel sensitivity	1
	Saturated Fat Sensitivity	3
	Sensitivity and response to particular foods	2
	Refined carbohydrate Sensitivity	1
**Macronutrients**		
	Lipid metabolism	13
	Carbohydrate metabolism	6
	Protein metabolism	1
	Whole grains metabolism	1
**Micronutrients**		
	Vitamin D metabolism	13
	Vitamin C metabolism	10
	Vitamin A metabolism	9
	Vitamina B6 metabolism	9
	Vitamin B12 metabolism	9
	Vitamin B9 metabolism	6
	Vitamina E metabolism	6
	Iron metabolism	4
	Vitamina B metabolism	4
	Calcium metabolism	2
	Metabolism of vitamins and minerals	1
	Sodium metabolism	1
	Vitamina F metabolism	1
	Vitamin K metabolism	1
**Eating behaviour**		
	Weight menagement	12
	Hunger and appetite control	4
	Regulation of metabolism and eating behavior	3
	Eating habits	1
	Food craving	1
	Feeling full	1
**Physiological parameters**		
	Levels of lipid profile	5
	Basal metabolism	1
	BMI	1
	Nutrient response	2
	Nutritional needs	3
	Blood pressure	1
	Microbiome	1
	Bitter taste	2
**Oxidative stress**		
	Antioxidant and detoxifying capacity	3
	Oxidative stress	1
	Antioxidant needs	1
	Glutathione	1
**Hormones**		
	Insulin sensivity	1
	Insulin resistance	1
	Risk of developing insulin resistance	1
**Other**		
	N.D. items	15

## Supplementary Materials

The following are available online at https://www.mdpi.com/2072-6643/12/2/566/s1: Table S1: table of traits analyzed by DTC-GT, Table S2: table of genes and variants of interest, Table S3: table of GWAS catalog results, Table S4: table of Clinvar results.

## References

[1] Crow D. (2019). A New Wave of Genomics for All. Cell.

[2] Webborn N., Williams A., McNamee M., Bouchard C., Pitsiladis Y., Ahmetov I., Ashley E., Byrne N., Camporesi S., Collins M. (2015). Direct-to-consumer genetic testing for predicting sports performance and talent identification: Consensus statement. Br. J. Sports Med..

[3] Zhang G., Nebert D.W. (2017). Personalized medicine: Genetic risk prediction of drug response. Pharmacol. Ther..

[4] Guasch-Ferré M., Dashti H.S., Merino J. (2018). Nutritional genomics and direct-to-consumer genetic testing: An overview. Adv. Nutr..

[5] International Human Genome Sequencing Consortium (2004). Finishing the euchromatic sequence of the human genome. Nature.

[6] dbSNP. https://www.ncbi.nlm.nih.gov/snp/.

[7] Sherry S.T., Ward M.H., Kholodov M., Baker J., Phan L., Smigielski E.M., Sirotkin K. (2001). dbSNP: The NCBI database of genetic variation. Horm. Res..

[8] Landrum M.J., Lee J.M., Riley G.R., Jang W., Rubinstein W.S., Church D.M., Maglott D.R. (2014). ClinVar: Public archive of relationships among sequence variation and human phenotype. Nucleic Acids Res..

[9] RefSNP Report. https://www.ncbi.nlm.nih.gov/snp/rs1801280.

[10] Barbarino J.M., Whirl-Carrillo M., Altman R.B., Klein T.E. (2018). PharmGKB: A worldwide resource for pharmacogenomic information. Wiley Interdiscip. Rev. Syst. Biol. Med..

[11] Nyaga D.M., Vickers M.H., Perry J.K., O’Sullivan J.M. (2018). The genetic architecture of type 1 diabetes mellitus. Mol. Cell. Endocrinol..

[12] Cuyvers E., Sleegers K. (2016). Genetic variations underlying Alzheimer’s disease: Evidence from genome-wide association studies and beyond. Lancet Neurol..

[13] Marchini J., Howie B. (2010). Genotype imputation for genome-wide association studies. Nat. Rev. Genet..

[14] Tam V., Patel N., Turcotte M., Bossé Y., Paré G., Meyre D. (2019). Benefits and limitations of genome-wide association studies. Nat. Rev. Genet..

[15] Buniello A., Macarthur J.A.L., Cerezo M., Harris L.W., Hayhurst J., Malangone C., McMahon A., Morales J., Mountjoy E., Sollis E. (2019). The NHGRI-EBI GWAS Catalog of published genome-wide association studies, targeted arrays and summary statistics 2019. Nucleic Acids Res..

[16] GWAS Catalog. https://www.ebi.ac.uk/gwas/.

[17] Zenin A., Tsepilov Y., Sharapov S., Getmantsev E., Menshikov L.I., Fedichev P.O., Aulchenko Y. (2019). Identification of 12 genetic loci associated with human healthspan. Commun. Biol..

[18] Costa V., Casamassimi A., Ciccodicola A. (2010). Nutritional genomics era: Opportunities toward a genome-tailored nutritional regimen. J. Nutr. Biochem..

[19] Loos R.J.F. (2019). From nutrigenomics to personalizing diets: Are we ready for precision medicine?. Am. J. Clin. Nutr..

[20] Wray N.R., Yang J., Hayes B.J., Price A.L., Goddard M.E., Visscher P.M. (2013). A commentary on Pitfalls of predicting complex traits from SNPs. Nat. Rev. Genet..

[21] Tenesa A., Haley C.S. (2013). The heritability of human disease: Estimation, uses and abuses. Nat. Rev. Genet..

[22] Moore D.S., Shenk D. (2016). The heritability fallacy. Wiley Interdiscip. Rev. Cogn. Sci..

[23] Schrempft S., van Jaarsveld C.H.M., Fisher A., Herle M., Smith A.D., Fildes A., Llewellyn C.H. (2018). Variation in the Heritability of Child Body Mass Index by Obesogenic Home Environment. JAMA Pediatr..

[24] Duncan L., Shen H., Gelaye B., Meijsen J., Ressler K., Feldman M., Peterson R., Domingue B. (2019). Analysis of polygenic risk score usage and performance in diverse human populations. Nat. Commun..

[25] Jordan B. (2018). Chroniques génomiques Balayage du génome des personnes à risque. Médecine/Sciences..

[26] Regalado A. (2019). More than 26 Million People Have Taken an At-Home Ancestry Test. Mit. Technol. Rev..

[27] Friend L., Rivilin A., O’Neil J., Browne R. Direct-to-Consumer Genetic Testing: Opportunities and Risks in a Rapidly Evolving Market.

[28] Wood L. Global Direct-to-Consumer Genetic Testing (DTC-GT) Market: Focus on Direct-to-Consumer Genetic Testing Market by Product Type, Distribution Channel, 15 Countries Mapping, and Competitive Landscape—Analysis and Forecast, 2019–2028.

[29] University College Dublin, National University of Ireland Dublin Final Report Summary—FOOD4ME (Personalised Nutrition: An Integrated Analysis of Opportunities and Challenges).

[30] Locke A.E., Kahali B., Berndt S.I., Justice A.E., Pers T.H., Day F.R., Powell C., Vedantam S., Buchkovich M.L., Yang J. (2015). Genetic studies of body mass index yield new insights for obesity biology. Nature.

[31] Loos R.J.F., Yeo G.S.H. (2014). The bigger picture of FTO—The first GWAS-identified obesity gene. Nat. Rev. Endocrinol..

[32] Yengo L., Sidorenko J., Kemper K.E., Zheng Z., Wood A.R., Weedon M.N., Frayling T.M., Hirschhorn J., Yang J., Visscher P.M. (2018). Meta-analysis of genome-wide association studies for height and body mass index in ~700 000 individuals of European ancestry. Hum. Mol. Genet..

[33] Ruiz-Ojeda F.J., Anguita-Ruiz A., Leis R., Aguilera C.M. (2018). Genetic factors and molecular mechanisms of Vitamin D and obesity relationship. Ann. Nutr. Metab..

[34] Basoli V., Santaniello S., Cruciani S., Ginesu G.C., Cossu M.L., Delitala A.P., Serra P.A., Ventura C., Maioli M. (2017). Melatonin and vitamin D interfere with the adipogenic fate of adipose-derived stem cells. Int. J. Mol. Sci..

[35] Cruciani S., Santaniello S., Garroni G., Fadda A., Balzano F., Bellu E., Sarais G., Fais G., Mulas M., Maioli M. (2019). Myrtus polyphenols, from antioxidants to anti-inflammatory molecules: Exploring a network involving cytochromes P450 and Vitamin D. Molecules.

[36] Santaniello S., Cruciani S., Basoli V., Balzano F., Bellu E., Garroni G., Ginesu G.C., Cossu M.L., Facchin F., Delitala A.P. (2018). Melatonin and vitamin D orchestrate adipose derived stem cell fate by modulating epigenetic regulatory genes. Int. J. Med. Sci..

[37] Jiang X., O’Reilly P.F., Aschard H., Hsu Y.H., Richards J.B., Dupuis J., Ingelsson E., Karasik D., Pilz S., Berry D. (2018). Genome-wide association study in 79,366 European-ancestry individuals informs the genetic architecture of 25-hydroxyvitamin D levels. Nat. Commun..

[38] Dudbridge F. (2013). Power and Predictive Accuracy of Polygenic Risk Scores. PLoS Genet..

